# Differential Surface Capping Effects on the Applications of Simple Amino-Acid-Capped ZnS:Mn Nanoparticles

**DOI:** 10.3390/mi12091064

**Published:** 2021-08-31

**Authors:** Jinwoo Park, Cheong-Soo Hwang

**Affiliations:** Department of Chemistry, Dankook University, Cheonan 31116, Korea

**Keywords:** ZnS:Mn nanoparticles, l-alanine capping, l-glycine capping, l-valine capping, photosensor, colloidal nanoparticles

## Abstract

Water-dispersible ZnS:Mn nanoparticles (NPs) were prepared by capping their surface with simple structured amino acids: l-alanine (Ala), l-glycine (Gly), and l-valine (Val) molecules, which have very similar structures except for the terminal organic functional groups. The detailed characterization works for the prepared colloidal NPs were performed using various spectroscopic methods. In particular, the NPs commonly showed UV/visible absorption peaks around 325 nm and PL emission peaks around 590 nm, corresponding to the wavelength of orange color light. In this study, these amino-acid-capped NPs were applied as optical photosensors in the detection of specific divalent transition metal cations in the same conditions. Consequently, all three NPs showed exclusive fluorescence quenching effects upon the addition of Cu (II) metal ions, whereas their quenching efficiencies were quite different to each other. These experimental results indicated that the Gly-ZnS:Mn NPs (k = 4.09 × 10^5^ M^−1^) can be the most effective optical photosensor for the detection of Cu^2+^ ions in water among the three NPs in the same conditions. This study showed that the steric effect of the capping ligand can be one of the key factors affecting the sensor activities of the ZnS:Mn NPs.

## 1. Introduction

Various surface modification methods for synthesizing water-soluble semiconductor nanocrystal materials are currently being intensively studied in various fields for various application purposes [1,2,3,4,5]. However, since most semiconductor nanocrystal materials have very low solubility in water, synthesizing well-dispersed colloidal nanoparticles in aqueous solution remains as a major challenge [6]. In addition, another serious problem that should be solved to directly apply these semiconductor materials to biological systems is that these semiconductor materials usually have high biological toxicity [7]. In order to reduce the cytotoxicity of the semiconductor nanoparticles, many attempts have addressed the development of novel types of polar and nontoxic capping ligands [8]. Among them, biocompatible amino-acid ligands have high solubility in water and low biological toxicity; thus, so they are expected to be appropriate surface capping ligands for the semiconductor nanomaterials for bio-related applications [9]. Therefore, research using various types of amino acids as a surface capping agent to synthesize colloidal nontoxic semiconductor nanoparticles has been conducted for the development of bioimaging agents for medical diagnosis such as malignant tumors or cancers [10]. In addition, recently, the field of application of nanoparticles capped with these amino acids has been greatly expanded not only in the bio-related research field, but also in the fields of optical sensors [11] and photocatalysts [12] using their unique electrochemical properties. We previously reported the synthesis of water-dispersible ZnS:Mn nanoparticles surface-capped with l-glycine and l-alanine to evaluate their biological toxicities over the growth of *E. coli* bacteria [13]. In that article, the ZnS parent crystals were chosen because they do not contain biologically and environmentally harmful elements such as Cd and Hg. In addition, they are also known to have sufficiently stable optical and physical properties in various (e.g., highly acidic or basic) conditions [14,15]. As a result, the Gly-ZnS:Mn and Ala-ZnS:Mn nanoparticles showed very low toxic effects on the growth of *E. coli* bacteria at ambient temperature with very high dispersibility in water. Therefore, the colloidal ZnS:Mn nanoparticles capped with these amino acids were considered as high-efficiency fluorescent biolabeling materials that can replace the highly toxic cadmium-based traditional semiconductor core materials. In this article, the synthesis and applications of the colloidal ZnS:Mn nanoparticles surface-capped with l-alanine (Ala), l-glycine (Gly), and l-valine (Val) are described. These three amino acids have almost identical molecular backbone structures, but they have different functional groups at the end of the molecules. Moreover, those colloidal nanoparticles were applied as optical photosensors to detect specific metal ion species in water in the same conditions. The ultimate purpose of this study was to compare the relative differences of the surface capping effect induced by three different but structurally similar amino-acid molecules on the optical sensor properties in terms of selectivity and sensitivity of the ZnS:Mn nanoparticles.

## 2. Materials and Methods

### 2.1. Experimental Procedures

The synthetic procedures of the water-dispersible Gly-ZnS:Mn, Ala-ZnS:Mn, and Val-ZnS:Mn nanoparticles were quite similar to that of the previously reported l-glycine-capped ZnS:Mn nanoparticles [13]. The major modifications were the adjustment of the pH of the colloidal solutions to highly basic (pH 10) for further application studies at the last step.

Therefore, all the experimental data were slightly different from those listed in the previous paper. First, 5.0 mmol of ZnSO_4_ solid (Sigma-Aldrich, St. Louis, MO, USA, Reagent grade) was dissolved in 25 mL of freshly distilled deionized water. In a different flask, 10 mmol of corresponding amino acid (l-Gly, l-Ala, or l-Val, Sigma-Aldrich, Reagent grade) solid was dissolved in 25 mL of deionized water and added to the Zn-containing aqueous solution. In this mixing process, a bath of an ice-salt mixed solution was used to keep the overall reaction temperature at 0–5 °C with stirring. In this process, formation of the [Zn–(amino acid)] precursor complex was induced. In another Pyrex glass flask, MnSO_4_ (Sigma-Aldrich, Reagent grade, 0.1 mmol) and Na_2_S (Sigma-Aldrich, Reagent grade, 5.0 mmol) solids were dissolved together in 30 mL of 1.0 × 10^−6^ M diluted sulfuric acid solution. This solution was transferred to the flask containing the [Zn–(amino acid)] precursor with continuous stirring, and then the entire solution was heated to reflux (about 90 °C). In this system, the calculated final molar concentrations for the essential components in the total solution were 6.3 × 10^−2^ M [Zn], 6.3 × 10^−2^ M [S], 1.3 × 10^−3^ M [Mn], and 1.3 × 10^−1^ M [l-amino acids]. After refluxing for about 20 h, the digital hot plate was removed and the nanoparticle-containing solution was allowed to cool naturally. Next, 100 mL of absolute ethanol was poured to induce the formation of white-gray precipitates at the bottom of the flask. The product nanoparticle powder was separated using a centrifugal separator (Sorvall ST8R, Wakenyaku Co.,Ltd., Kyoto, Japan, 30,279× *g*), and then the solution of the upper layer was decanted. The off-white powder was quickly washed with a cold ethanol-–water (8:2) mixture and dried in a vacuum oven overnight (about 60 °C). Finally, the powder of the nanoparticles was dissolved in deionized water, and a 1.0 × 10^−6^ M dilute NaOH solution was slowly and carefully added until the pH of the total solution reached 10. The essential experimental data of colloidal Gly-ZnS:Mn, Ala-ZnS:Mn, and Val-ZnS:Mn nanoparticles obtained under this condition are summarized in Table 1.

### 2.2. Instrumentation

**Figure 1:**
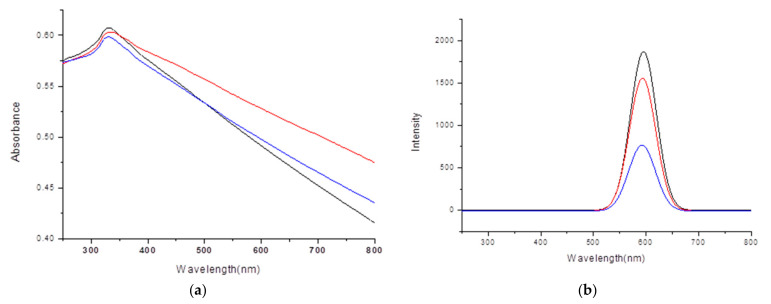
(**a**) UV visible absorption spectra of Gly-ZnS:Mn (black), Ala-ZnS:Mn (red), and Val-ZnS:Mn (blue) NPs. (**b**) Photoluminescence (PL) emission spectra of Gly-ZnS:Mn (black), Ala-ZnS:Mn (red), and Val-ZnS:Mn (blue) NPs.

**Figure 2:**
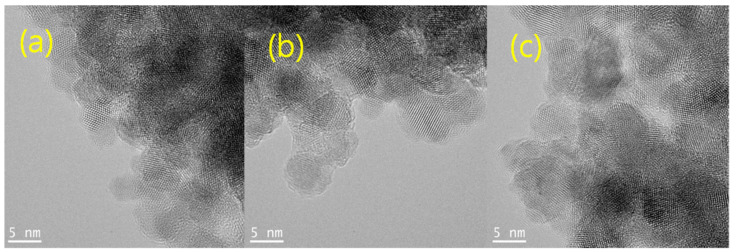
HR-TEM images of: (**a**) Gly-ZnS:Mn, (**b**) Ala-ZnS:Mn, and (**c**) Val-ZnS:Mn NPs.

**Figure 3:**
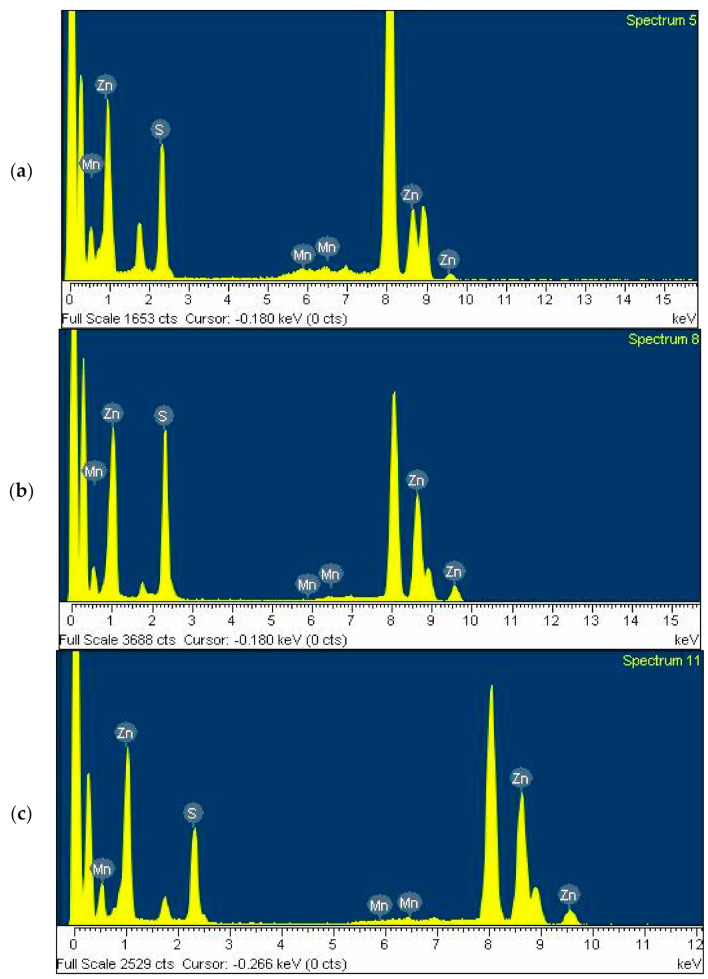
EDXS diagrams of (**a**) Gly-ZnS:Mn, (**b**) Ala-ZnS:Mn, and (**c**) Val-ZnS:Mn NPs.

**Figure 4:**
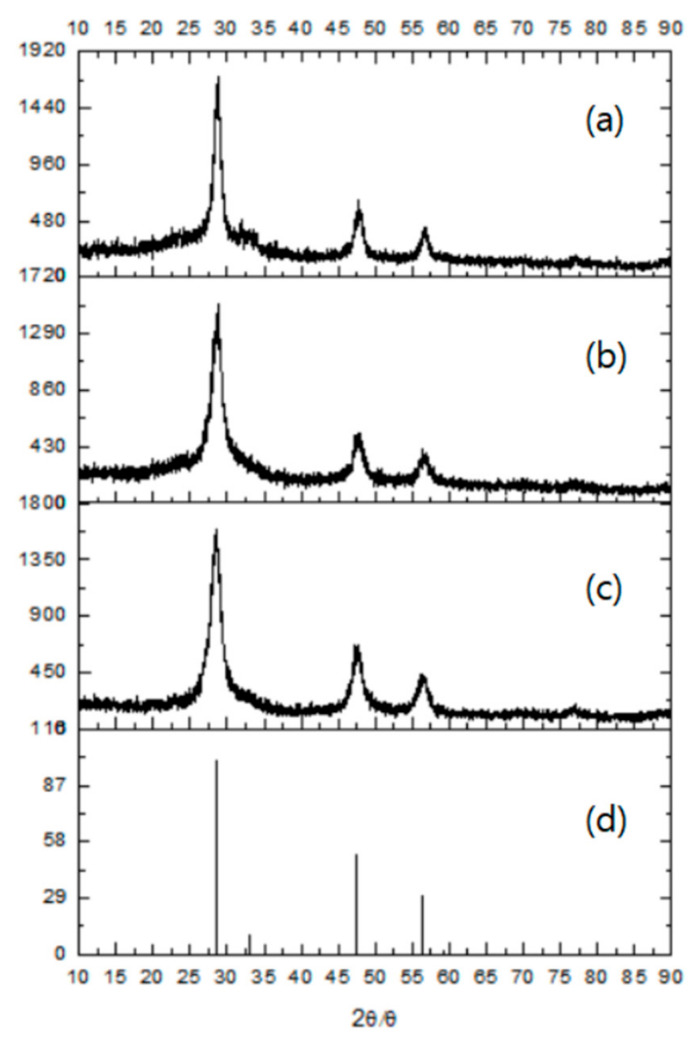
XRD pattern diagrams of (**a**) Gly-ZnS:Mn, (**b**) Ala-ZnS:Mn, and (**c**) Val-ZnS:Mn NPs, and (**d**) a reference bulk ZnS solid in a zinc blende phase.

**Figure 5:**
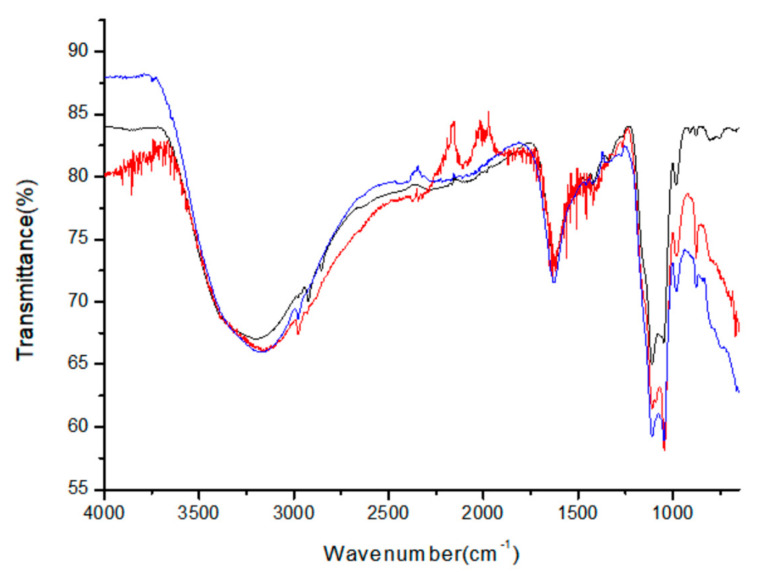
FT-IR spectra of Gly-ZnS:Mn (black), Ala-ZnS:Mn (red), and Val-ZnS:Mn (blue) NPs.

The UV/visible spectra in Figure 1a were obtained using a Lamda-25 spectrophotometer (Perkin-Elmer, Waltham, MA, USA), and the room-temperature solution photoluminescence (PL) spectra in Figure 1b were obtained using an LS-45 spectrophotometer (Perkin-Elmer) equipped with a 500 W xenon lamp as a light source. The presented pictures of high-resolution-transmission electron microscopy (HR-TEM in Figures 2 and 3) were obtained using a JEOL JEM 1210 electron microscope, in which the magnification range was from 1000 to 800,000, and the accelerating voltage was from 40 to 120 kV. The powder X-ray diffraction (XRD) pattern diagrams of the NPs presented in Figure 4 were obtained using an X-ray diffractometer, Rigaku 300, which used a Cu-Kα (wavelengths of 1.54 Å) X-ray light source. The elemental analyses for the Gly-ZnS:Mn, Ala-ZnS:Mn, and Val-ZnS:Mn nanoparticles were performed using a Perkin-Elmer Optima-430 ICP-AES spectrometer. The surface capping ligands, i.e., amino acids (Gly, Ala, and Val), were investigated in terms of their specific vibrational modes using Fourier-transform infrared spectroscopy (FT-IR, in Figure 5) recorded using a Spectrum One spectrophotometer (Perkin-Elmer, resolution of 1.0 cm^−1^) having an attenuated total reflection (ATR) unit. The number of scans was set to 32 for the liquid samples of the NPs.

## 3. Results and Discussions

### 3.1. Characterizations of the Gly-ZnS:Mn, Ala-ZnS:Mn, and Val-ZnS:Mn Nanoparticles

The specific optical properties of the synthesized Gly-ZnS:Mn, Ala-ZnS:Mn, and Val-ZnS:Mn nanoparticles were determined using UV/visible spectroscopy (Figure 1a) and photoluminescence spectroscopy (PL, Figure 1b). The absorption spectrum shows the energy differences between the valence and the conduction bands formed in the ZnS parent nanocrystals [16]; this observed average band gap of the ZnS:Mn nanocrystals capped with three amino acids was 3.84 eV, whereas that of the bulk ZnS:Mn solid was 3.54 eV. This was probably due to the well-known quantum confinement effect for nanoscale semiconductor materials [17].

In the PL spectra of the Gly-ZnS:Mn, Ala-ZnS:Mn, and Val-ZnS:Mn nanoparticles, broad emission peaks at 590 nm were found in common. The provided PL spectra were obtained by setting the excitation wavelength of the spectrophotometer light source to the wavelengths of the UV/visible absorption peaks obtained from the nanoparticle colloids, which were 328 nm (Gly-ZnS:Mn), 323 nm (Ala-ZnS:Mn), and 328 nm (Val-ZnS:Mn). As one can see in those PL spectra, the peak positions of the Gly-ZnS:Mn, Ala-ZnS:Mn, and Val-ZnS:Mn nanocrystals were almost identical, but the intensity of the PL peaks were quite different. The Gly-ZnS:Mn nanoparticles presented much higher intensities than other ZnS:Mn nanocrystals. The emission peak at about 590 nm for the ZnS:Mn nanocrystals is a result of the dopant metal cation (Mn^2+^) and is often expressed as a ^4^T_1_–^6^A_1_ transition [18].

The large Stokes shift (about 300 nm) which appeared between the absorption and emission wavelengths for the amino-acid-capped colloidal ZnS:Mn nanoparticles was probably caused by the energy levels of the surface defects, which were placed close enough to the conduction band of the ZnS parent crystals [19]. This observed phenomenon is one of the typical and characteristic features of most semiconductor nanocrystalline materials doped with transition metal ions such as Mn^2+^ [20].

The relative PL quantum efficiencies of the prepared colloidal Gly-ZnS:Mn, Ala-ZnS:Mn, and Val-ZnS:Mn nanoparticles were experimentally measured and calculated by following the exact same procedures reported by Williams et al. [21]. This method calculates the relative quantum efficiency of ZnS:Mn nanocrystals capped with amino acids by comparing to a well-known standard reference material which has a very similar emission wavelength to that of the ZnS:Mn nanoparticles in the literature [16]. In this study, 0.1 M H_2_SO_4_ aqueous solution containing quinine sulfate (Fluka) was used as a reference material, which is known to have an absolute quantum efficiency of 54.6% at 22 °C [22]. In these experiments, the excitation wavelength for the reference dye (quinine sulfate) was fixed at the same UV/visible absorption wavelengths observed for each amino-acid-capped ZnS:Mn nanoparticles. The PL spectra for corresponding amino-acid-capped ZnS:Mn nanoparticles and the reference dye were taken at five different molar concentrations. Then, diagrams of the absorbance vs. the integrated PL intensity for both samples were plotted together. Finally, the relative quantum efficiencies for the colloidal Gly-ZnS:Mn, Ala-ZnS:Mn, and Val-ZnS:Mn nanoparticles against the reference dye were calculated using the following equation:

(1) $$Qa=Qr\left(\frac{Grad_{a}}{Grad_{r}}\;\right)\left(\;\frac{\varepsilon_{a}^{2}}{\varepsilon_{r}^{2}}\;\right),$$

where Q is quantum yield, and ‘r’ and ‘a’ represent the reference (quinine sulfate dye) and the amino-acid-capped ZnS:Mn nanoparticles, respectively. Moreover, ‘ε’ and ‘Grad’ are the gradient and the refractive index of the solvent used in these experiments, respectively. In fact, the solvent factor can be eliminated by carrying out the analysis using the same solvent (water) for the reference and the colloidal nanoparticle samples. The resulting relative quantum efficiencies were 6.3% (Gly-ZnS:Mn), 4.1% (Ala-ZnS:Mn), and 3.0% (Val-ZnS:Mn). These were similar to or slightly higher than ZnS:Mn nanoparticles capped with other amino-acid ligands [13,14]. Among them, the colloidal Val-ZnS:Mn nanoparticles had the highest quantum efficiency, which was probably caused by the fact that l-valine molecules do not have sterically bulk substituents; hence, the capping molecules could be placed more closely to the nanoparticle surface. This could be a very important factor in improving the optical properties [23].

In order to compare the relative optical properties for the three different ZnS:Mn NPs, we analyzed their UV/visible and PL spectra in the same concentration conditions. However, most generally known methods in the literature for the concentration determination of certain nanoparticles using various methods, such as UV/visible spectroscopy [24], ICP-MS [25], and HR-TEM [26], have a critical problem in that they cannot actually simultaneously evaluate the concentrations of organic capping molecules. In this study, the volume and the mass of the single nanoparticle of each amino-acid-capped ZnS:Mn NP were calculated using the particle sizes measured in the HR-TEM pictures and the known density for bulk ZnS:Mn solid (4.09 g/cm^3^), assuming that the nanoparticles held the same density as the bulk solid and the shapes of the NPs were perfectly spherical. Using this method, we calculated the number of particles in 10 mL of the colloidal ZnS:Mn NP solutions. Even though this method cannot cover the concentrations of the capping amino=acid molecules, it can still provide useful information since the absorptions and emissions of light by the NPs are mainly contributed by the semiconductor parent crystals. In addition, these calculations very strongly depend on the size of the NPs; thus, the obtained NP concentrations for Gly-ZnS:Mn, Ala-ZnS:Mn, and Val-ZnS:Mn NPs were very close to each other (ca. 2.1 × 10^15^ NPs/mL in the solution containing 10 mg of the NPs) because they have very similar particle sizes in the solid state, as shown in their HR-TEM images. Nevertheless, those resulting PL spectra showed very different intensities and integrated peak areas for the emission peaks for the NPs, directly related to their quantum yields, which can be also affected by the surface capping ligands.

The particle sizes of the Gly-ZnS:Mn, Ala-ZnS:Mn, and Val-ZnS:Mn nanoparticles in the solid state were measured directly from the images obtained using high-resolution transmission electron microscopy (HR-TEM) provided in Figure 2. The average particle sizes of the nanocrystals were obtained by measuring about 20 identifiable spherical particles along the fringe images in each picture. As a result, the obtained average particle sizes were 6.6 ± 0.7 nm (Gly-ZnS:Mn) 6.8 ± 0.9 nm (Ala-ZnS:Mn), and 6.7 ± 1.1 nm (Val-ZnS:Mn). It can be distinctly seen that the product nanoparticles were made of a single crystalline material, not a simple mixture of their precursors, according to the obvious fringe images with an interstitial spacing of about 0.3 nm. The average particle sizes of these nanoparticles were very similar to that of ZnS:Mn nanocrystals capped with other organic ligands (average 5.0 nm) [18,19,20]. However, as shown in the HR-TEM pictures, some aggregation between nanoparticles occurred, making it difficult to identify and measure the size of the individual nanoparticles. This can be attributed to the agglomeration that occurred during the rapid drying process using a high-vacuum oven to prepare the samples for the HR-TEM measurements. The NP samples for the HR-TEM analyses were prepared using carbon-coated copper grids (300 Mesh). The solid NP powders were redispersed in a water/ethanol mixture solution and placed on the copper grid, followed by drying in a high-vacuum oven at 80 °C for 10 h.

Figure 3 shows the energy-dispersive X-ray spectroscopy (EDXS) diagrams obtained from the Gly-ZnS:Mn, Ala-ZnS:Mn, and Val-ZnS:Mn nanoparticles. These diagrams show that all nanoparticle samples were made of Zn, S, and Mn elements, and their relative at.% ratios were 68.1:36.0:1.6 (Gly-ZnS:Mn), 69.9:33.7:0.6 (Ala-ZnS:Mn), and 66.1:34.2:1.2 (Val-ZnS:Mn). However, inductively coupled plasma atomic emission spectroscopy (ICP-AES) analyses were also performed to more precisely determine the concentration of the dopant Mn^2+^ ions in the ZnS:Mn nanoparticles capped with the three amino acids. In this study, we tried to control the concentration range of the dopant Mn ions within 1–2% of the ZnS parent, as this has been described in the literature as a condition that shows optimal fluorescence quantum efficiency for the ZnS:Mn nanocrystals capped with many other organic ligands [18]. In this study, the actually measured at.% concentrations of the Mn^2+^ ions were 1.1 (Gly-ZnS:Mn), 0.8 (Ala-ZnS:Mn), and 1.0 (Val-ZnS:Mn), indicating that the concentrations of the dopant Mn ions were well controlled as intended.

Figure 4 presents the X-ray diffraction (XRD) pattern diagrams recorded from powder samples of Gly-ZnS:Mn, Ala-ZnS:Mn, and Val-ZnS:Mn nanoparticles and bulk ZnS solids as a reference. The XRD pattern diagrams showed broad peaks, which is one of the well-known typical properties of nanometer-sized semiconductor materials in the solid state [27]. Nevertheless, it is shown that the three peaks in the diagram, known as (111), (220), and (311) planes, correspond exactly to those of the previously known cubic zinc blend crystal structure (JCPDS 05-0566). Therefore, it can be seen that Gly-ZnS:Mn, Ala-ZnS:Mn, and Val-ZnS:Mn nanocrystals all have the parent crystal structure of the cubic zinc blend, which is the kinetically preferred form in the solid state [28]. In addition, the particle sizes of Gly-ZnS:Mn, Ala-ZnS:Mn, and Val-ZnS:Mn nanoparticles were calculated using the obtained XRD peak data and the Debye–Scherrer formula for comparison with the values measured in the HR-TEM images [29]. As a result, the calculated average particle sizes of Gly-ZnS:Mn, Ala-ZnS:Mn, and Val-ZnS:Mn nanoparticles were 7.1 nm, 6.9 nm, and 6.8 nm, respectively. The calculated sizes of the nanoparticles were very close to those directly measured from the HR-TEM images, also confirming that they were made of single crystalline materials.

The specific vibrational and coordination modes of the capping amino-acid molecules were assigned and characterized using FT-IR (Fourier-transform infrared) spectroscopy to experimentally determine whether the amino-acid ligands are actually attached to the surface of the ZnS:Mn nanoparticles. Figure 5 shows the superimposed FT-IR spectra obtained from the colloidal Gly-ZnS:Mn, Ala-ZnS:Mn, and Val-ZnS:Mn nanoparticles. The obtained FT-IR peaks were analyzed by comparison with the reference data for each amino-acid molecule in a free state (not capped on the nanoparticles) [30,31,32].

The most characteristic peaks of the surface-capped amino acid molecules in each FT-IR spectrum were the two carbonyl (C=O) stretching peaks at 1550 and 1450 cm^−1^. This carbonyl stretching peak is very different from the peak (1700 cm^−1^) of the corresponding amino-acid molecule in the free state, strongly suggesting that the carbonyl group is coordinated to the Zn^2+^ or Mn^2+^ ions on the surface of the nanocrystals. Moreover, the broad N–H stretching peak around 3000 cm^−1^ is very similar to the peak of the uncoordinated amino-acid molecule. Therefore, it can be concluded that, in the amino-acid molecule, the carbonyl group is actually coordinated to the surface of the nanoparticles, whereas the amine group remains free in aqueous solution. Other bands appearing in the FT-IR spectra for the three amino-acid molecules, which were coordinated directly to the surface of ZnS:Mn nanocrystals, were assigned as follows: O–H and N–H stretching peaks (3550–3250 cm^−1^), symmetric and asymmetric C–H stretching peaks (2990–2880 cm^−1^), C–O–H bending vibration peak (1435–1220 cm^−1^), C–C and C–N bond stretching vibration peak (1120–890 cm^−1^), and C–C and C–O bond bending vibration peak (890–350 cm^−1^). Additional specific peak data and their assignments based on the corresponding references are provided in Table 2.

Even though it was hard to structurally distinguish them on the basis of their spectroscopical characterizations, it is well known that the steric effects induced by the side groups of these three l-amino acids are quite different. For instance, the differential steric effects induced by the side groups in l-glycine and l-valine on a proton (H^+^) transfer reaction of these amino acids have been reported in the literature [33]. According to the authors’ descriptions, the bulky isopropyl side group in the valine molecule can drastically decrease the proton transfer reaction rate of the valine molecules, compared to that of glycine, due to the increased steric hinderance at the active center of the molecule, which was induced by the neighboring freely rotating isopropyl group at the same conditions. Therefore, it would be a reasonable assumption that the side groups in the amino-acid molecules attached to the surface of the NPs can similarly affect the ionic collision process between the NPs and the approaching metal ion species in water.

### 3.2. Photosensor Applications of the Gly-ZnS:Mn, Ala-ZnS:Mn, and Val-ZnS:Mn Nanoparticles

**Figure 6:**
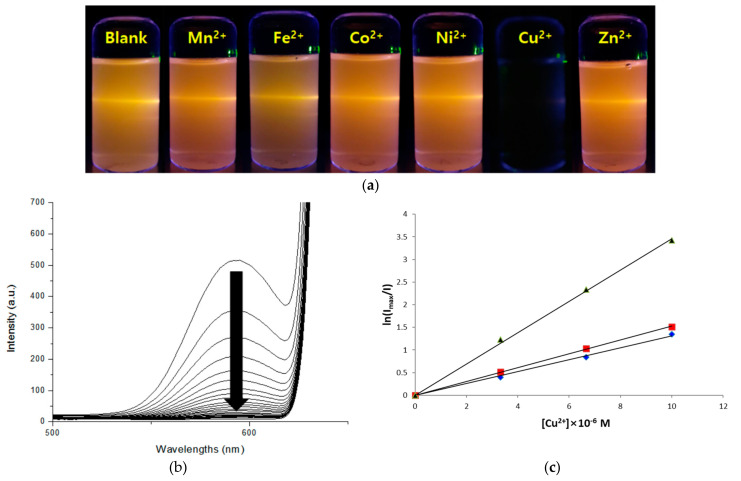
(**a**) Fluorescence images of the Gly-ZnS:Mn NPs upon addition of corresponding divalent transition metal ions, taken under irradiation of a UV lamp (325 nm). ‘Blank’ refers to NPs only. (**b**) PL spectral changes of the Gly-ZnS:Mn NPs upon addition of a Cu^2+^ ion-containing solution. (**c**) Linear fitting diagrams showing the Stern–Volmer relationship between the PL intensity changes and the concentrations of the added Cu(II) ions to the Gly-ZnS:Mn (black), Ala-ZnS:Mn (red), and Val-ZnS:Mn (blue) NPs.

The synthesized Gly-ZnS:Mn, Ala-ZnS:Mn, and Val-ZnS:Mn colloidal nanoparticles were applied as optical sensors for the detection of specific ionic species among the first-row transition metal cations in aqueous solution. Figure 6a shows representative fluorescence images of the colloidal Gly-ZnS:Mn nanocrystals when aqueous solutions containing various divalent transition metal cations were added in the same conditions, such as concentration, pH, and temperature. These photos were taken using a 6 W UV lamp (325 nm, UV-Tech Inc., Chennai, India), with a light source having a wavelength similar to the absorption wavelength of the Gly-ZnS:Mn nanoparticles. As can be seen from the pictures, the orange fluorescence of the Gly-ZnS:Mn nanoparticles was maintained by the addition of most of metal ions, whereas the original fluorescence light of the nanocrystals was almost completely extinguished when Cu^2+^ ions were added. The reason why only the Gly-ZnS:Mn nanoparticle results are provided is that the other two nanoparticles also showed the same metal ion selectivity in the same conditions. In addition, Figure 6b shows the emission peak changes in the PL spectra of the colloidal Gly-ZnS:Mn nanocrystals when the concentration of copper(II) metal ions was gradually increased with respect to the initial colloidal solution of the nanoparticles. In these spectra, the intensity of the initial emission peak in the Gly-ZnS:Mn nanocrystals gradually decreased upon adding Cu^2+^ ions; ultimately, the peak completely disappeared (more than 98% quenched). As mentioned above, all three amino-acid-capped ZnS:Mn nanocrystals showed the same metal ion selectivity with exclusive fluorescence quenching by copper(II) ions. Therefore, the colloidal Gly-ZnS:Mn, Ala-ZnS:Mn, and Val-ZnS:Mn nanoparticles are very convenient and efficient optical sensor materials for detecting the presence of Cu^2+^ ions in liquid-phase food or environmental wastewater samples containing heavy transition metals. The detection of copper ions is important because copper ions are one of the most essential and fundamental elements of human life. For example, it is known that poor control of the concentration of copper ions in the body can cause serious diseases such as Menkes or Parkinson’s disease [34].

The limits of detection of the molar concentration [M] of copper (II) metal ions using ZnS:Mn nanoparticles capped with amino acids were 1.8 × 10^−7^ M (Gly-ZnS:Mn), 1.5 × 10^−7^ M (Ala-ZnS:Mn), and 1.1 × 10^−7^ M (Val-ZnS:Mn), and the concentration of the nanocrystals was 10.0 mg/L in all cases. The ion selectivity as an optical sensor of the ZnS:Mn nanocrystals capped with these three amino acids was completely different from that of the ZnS:Mn nanocrystals capped with structurally similar organic molecules such as mercaptoacetic acid (MAA) [35]. In the case of the MAA-ZnS:Mn nanocrystals, the addition of Zn^2+^ metal ions did not affect the fluorescence light, whereas all other divalent transition metal ions almost completely quenched the orange fluorescence light of the nanocrystals. According to the paper, the initial step of Zn^2+^ ion sensing using the colloidal MAA-ZnS:Mn nanocrystals was that positively charged transition metal ions were attracted to the negatively charged carbonyl groups of the surface capping MAA molecules attached to the nanocrystal surface, and then the adjacent ZnS:Mn nanocrystals formed metal bridging complexes such as [NP–COO–M^2+^–OOC–NP] in the solution. Lastly, electron transfer from the ZnS:Mn nanocrystals to the cross-linked transition metal ions occurred, resulting in fluorescence quenching for most metal ions. This is because most divalent transition metal ions have partially electron-filled orbitals in their valence shells. However, Zn^2+^ ions cannot accept electrons transferred from the nanoparticles because they already have a *d*_10_ electron configuration, fully filled with electrons. Therefore, the fluorescence of the ZnS:Mn nanocrystals does not decrease. Since our amino-acid-capped ZnS:Mn nanoparticles showed completely different metal ion selectivity compared to the MAA-ZnS:Mn nanoparticles, the quenching mechanism for the amino-acid-capped ZnS:Mn nanocrystals should be significantly different from that case.

As a similar example, Bo et al. published a research result showing that the CdTe nanocrystals capped with mercaptopropionic acid (MPA) showed a quenching effect only when Cu^2+^ ions were added [36]. Their kinetic studies suggested that the quenching mechanism is initiated by ionic collisions between Cu^2+^ ions and the negatively charged MPA-CdTe nanocrystals in aqueous solution. Another similar case is the exclusive quenching effect of the thioglycerol-capped CdS nanoparticles by the addition of Cu^2+^ ions, reported by Chen et al. [37]. In this paper, the authors provided critical experimental evidence for the electron transfer between Cu metal ions and nanocrystals using electron paramagnetic resonance (EPR) spectroscopy. It was shown that Cu^2+^ (*d*^9^) ions, which were initially paramagnetic, were reduced to diamagnetic Cu^1+^ (*d*^10^) species after collision with CdS nanocrystals. Comparing these cases, it can be inferred that the ZnS:Mn nanoparticles capped with the amino acids also collided similarly to the added Cu^2+^ ions in solution, while electron transition occurred, resulting in quenching of fluorescence. In addition, the plausible explanation that only the Cu^2+^ ions exhibit the fluorescence-quenching effect may be that one equivalent of electron transfer occurred between the nanoparticles and the added metal ions; therefore, the divalent ions were reduced to monovalent cations. Since the stable oxidation state of +1 is hardly known for most transition metal complexes outside of copper ions in water, it can be inferred that the actual stable electron transition between nanoparticles and the most transition metal ions rarely occur outside of copper ions [38]. In addition, the reason that capping with l-glycine molecules showed the best efficiency among the three types of amino acids is that, in the case of alanine or valine, there is a methyl or iso-propyl group that can freely rotate in solution; thus, the collision between approaching metal cations and nanoparticles is significantly interrupted by these steric functional groups. Therefore, the glycine molecule without such a functional side group can relatively easily interact with the added metal ions to raise the sensitivity of the ZnS:Mn nanoparticles as an optical sensor in aqueous solution.

To experimentally verify the speculated quenching mechanism, a Stern–Volmer kinetic experiment was performed to measure the degree of emission quenching of the Gly-ZnS:Mn nanoparticles, i.e.,, the attenuation of the PL peak intensity according to the increase in the concentration of added Cu^2+^ ions [39]. As a result, as the concentration of Cu^2+^ ions gradually increased, and the decay rate of the fluorescence of the Gly-ZnS:Mn nanoparticles fitted well with the natural log dependent Stern–Volmer equation as shown in Figure 6c. This figure shows a good linear relationship at concentrations between 0 and 10 µM (*R^2^* = 0.992). In this experiment, the concentration range of the quencher was carefully set so that the fluorescence was not completely quenched. The obtained rate constant values of the extinction were calculated from the slope of the drawn lines, which were Gly-ZnS:Mn (k = 4.09 × 10^5^ M^-1^), Ala-ZnS:Mn (k = 2.13 × 10^5^ M^−1^), and Val-ZnS:Mn (k = 1.98 × 10^5^ M^−1^), respectively. The diagrams in Figure 6c also strongly suggest that the fluorescence-quenching mechanism of the amino-acid-capped colloidal ZnS:Mn nanoparticles probably proceeds through ionic collisions between the negatively charged ZnS:Mn nanoparticles and the positive Cu^2+^ ions because the result of the above kinetic study was quite similar to that seen for other previously mentioned semiconductor nanoparticles with Cu^2+^ ions, which were proven to take the ionic collision pathway in their fluorescence-quenching mechanisms [36,37].

Comparing the quenching rate constants calculated in this experiment with the results of other semiconductor nanoparticles capped with other organic ligands, the sensing efficiencies of the Gly-ZnS:Mn, Ala-ZnS:Mn, and Val-ZnS:Mn nanoparticles were quite similar. However, one of the advantages of simple amino-acid capping for the ZnS:Mn nanoparticles over other organic ligands is that these amino acids have much lower cytotoxicity. Moreover, the ZnS:Mn nanoparticles do not contain biologically and environmentally hazardous components compared to the CdSe nanoparticles, which makes the amino-acid-capped ZnS:Mn nanoparticles suitable for direct biomedical or environmental sensor applications.

There have been some publications reporting very similar fluorescence quenching effects but proposing quite different mechanisms for the semiconductor nanoparticles upon the addition of transition metal ions. For instance, the CdSe/ZnS core–shell nanoparticles capped with bovine serum albumin (BSA) showed a similar exclusive fluorescence quenching effect upon addition of Cu^2+^ ions in aqueous solution [40]. In this article, according to the authors’ descriptions, the fluorescence quenching of the BSA-CdSe/ZnS nanoparticles upon the addition of Cu(II) ions was mainly caused by the formation and growth of additional copper sulfide (CuS) crystals on the surface of the nanoparticles. They claimed that the added Cu^2+^ ions (73 pm, ionic radius) are small enough to penetrate between the bulky BSA ligands to attack sulfide (S^−^) ions on the surface (ZnS shell layer) of the nanoparticles to form smaller CuS crystals. Eventually, the surface-grown CuS crystals can destroy the CdSe/ZnS core–shell nanoparticles due to a severe mismatch between the two crystal layers. In addition, the formed CuS crystals have extremely low solubility in water; therefore, they can induce precipitation of the whole CdSe/ZnS, CuS nanocomposites at the bottom of the flask, which rapidly induced the fluorescence quenching of the BSA-CdSe/ZnS nanoparticles. However, we do not agree that this case can apply to the amino-acid-capped ZnS:Mn nanoparticles because there is no experimental evidence indicating the formation of the CuS crystals on the surface of the amino-acid-capped ZnS:Mn nanoparticles. For instance, we did not find any difference in the XRD pattern diagrams taken after the addition of Cu^2+^ ions to these amino-acid-capped ZnS:Mn nanoparticles. Therefore, we conclude that this quenching mechanism for the BSA-CdSe/ZnS nanoparticles cannot explain the fluorescence quenching of the amino-acid-capped ZnS:Mn nanoparticles by copper ions.

**Figure 7:**
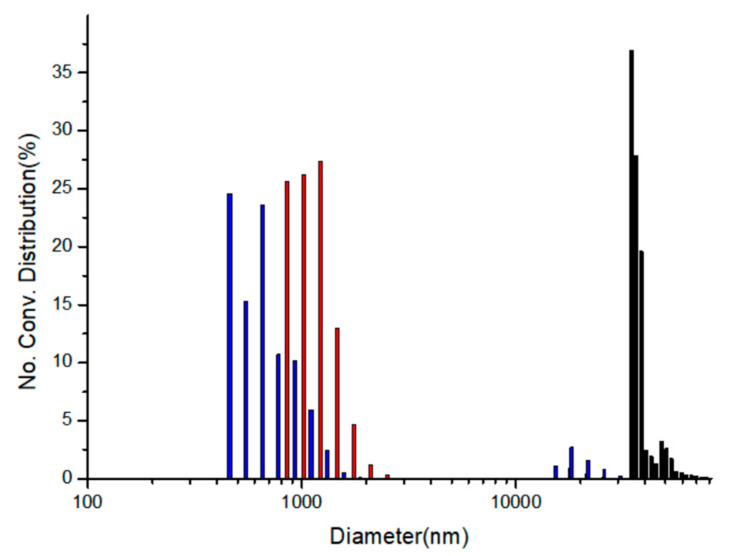
Size distribution diagrams of the colloidal Gly-ZnS:Mn (black), Ala-ZnS:Mn (red), and Val-ZnS:Mn (blue) NPs after addition of 4.0 × 10^−6^ M of Cu(II) ions.

Lastly, the degree of agglomeration of the amino-acid-capped ZnS:Mn nanoparticles after the addition of copper ions was compared to evaluate the relative capping effect differences among the Gly-ZnS:Mn, Ala-ZnS:Mn, and Val-ZnS:Mn nanoparticles in aqueous solution. Figure 7 shows the particle size distribution diagrams taken after the addition of copper ions to those corresponding nanoparticles using a particle size analyzer (Otsuka ELSZ-2000 spectrophotometer, Otsuka Electronics Co., Ltd., Osaka, Japan). In the diagram, 7 nm ZnS:Mn nanoparticles were originally grown to huge aggregates after the addition of 4.0 × 10^−6^ M of copper(II) ion-containing aqueous solutions. However, the degree of agglomeration of the nanoparticles was obviously different even though the concentrations of the added copper ions were exactly same. The average sizes of the aggregates were 35,890 nm (Gly-ZnS:Mn), 1108 nm (Ala-ZnS:Mn), and 787 nm (Val-ZnS:Mn nanoparticles). As can be seen in the diagram, l-glycine capping (with the least steric hinderance) caused the highest degree of agglomeration, being approximately 36 times larger than the other cases. These agglomerations were mainly caused by the electrostatic interactions between Cu^2+^ ions and the amino-acid-capped ZnS:Mn NP surface. However, the freely rotating functional side groups (methyl and isopropyl groups) in the l-alanine and the l-valine molecules significantly interrupted the aggregation process of the nanoparticles. They could strongly repel the incoming neighboring NPs via van der Waals repulsion. Therefore, the steric hinderance of the capping molecules can induce significant variations in the sensor activities and the agglomeration process for amino-acid-capped ZnS:Mn nanoparticles in aqueous solution. It is worth noting that we did not consider the aggregation formation process as a main factor for the fluorescence quenching of the ZnS:Mn NPs by the addition of the metal ions since, when we added same amount of Zn^2+^ ions to the colloidal amino-acid-capped NPs, we observed that similar aggregates were formed in the solution, but the yellow-orange florescence light from the NPs was not quenched at all.

Lastly, the calculated LOD values suggested that the Val-ZnS:Mn NPs may be more sensitive than the Gly-ZnS:Mn NPs in the sensing concentration range of ca. 10^−7^ M of copper ions. However, comparing the quenching rate constants, the Gly-ZnS:Mn NPs showed much higher values than the Val-ZnS:Mn NPs. This indicates that the degree of quenching for the Gly-ZnS:Mn NPs by the addition of the same amount of copper ions was much higher (more than double) than others. Therefore, in view of the research goal to develop a practical optical sensor for the detection of copper ions using the NPs, the Gly-ZnS:Mn NPs are much more useful. As such, we suggest the Gly-ZnS:Mn NPs as the best optical sensor materials among the three amino-acid-capped ZnS:Mn NPs. In addition, the reason that the Val-ZnS:Mn NPs had the lowest LOD value while the quenching rate constant was lower than that of the Gly-ZnS:Mn NPs can be explained according to Figure 7. The degree of aggregation of the NPs by the addition of the same amount of copper ions was the lowest for the Val-ZnS:Mn NPs. Therefore, the surface area/volume ratio for the Val-ZnS:Mn NPs was the highest among the three amino-acid-capped ZnS:Mn NPs during the sensing process, which could also affect the sensitivity of the NPs.

## 4. Conclusions

In this article, water-dispersible ZnS:Mn nanoparticles were synthesized using three conventional amino-acid molecules with similar structures but different functional groups, thus inducing different steric effects in the surface capping ligands. They were applied as selective and efficient optical sensor materials to detect specific transition metal ion species. The Gly-ZnS:Mn, Ala-ZnS:Mn, and Val-ZnS:Mn nanoparticles commonly showed an exclusive fluorescence quenching effect when Cu^2+^ ions were added. However, the quenching efficiency of the nanoparticles was quite different due to the different steric effects involving the capping ligands of the ZnS:Mn nanoparticles. As a result, among the three colloidal nanoparticles, the Gly-ZnS:Mn nanoparticles showed the best results in terms of optical sensor efficiency. This study clearly showed that the optical sensor efficiencies can vary greatly, even for semiconductor nanocrystals of the same parent, depending on the type of ligand molecule capped on the surface of the nanoparticles.

## Figures and Tables

**Table 1:** Data summary of the amino-acid-capped ZnS:Mn NPs.

	Gly-Zns:Mn	Ala-Zns:Mn	Val-ZnS:Mn
UV/Vis absorption (λ_max_, nm)	328	323	328
PL emission (λ_max_, nm)	591	589	591
PL efficiencies (%)	6.3	4.1	3.0
Conc.Mn dopant (%)	1.1	0.8	1.0
Average Particle Size HR-TEM (nm)	6.6	6.8	6.7

**Table 2:** FT-IR peak data and their assignment for Gly-ZnS:Mn, Ala-ZnS:Mn, and Val-ZnS:Mn NPs.

Gly-ZnS:Mn	Ala-ZnS:Mn	Val-ZnS:Mn	Assignments
326	359	361	C-C bending
488	497	489	C-C bending
590	602	605	C-C bending
686	697	690	C-C-O bending
897	892	890	C-C-N bending
1020	1015	1016	C-N bending
*1128*	*1125*	*1119*	C-C-O stretching
*1140*	*1138*	*1135*	C-N stretching
*1325*	*1325*	*1330*	C-C stretching
*1405*	*1411*	*1410*	C-O-H bending
*1460*	*1455*	*1467*	C=O stretching
*1558*	*1567*	*1550*	C=O stretching
*2990*	*2982*	*2980*	C-H stretching
*3305*	*3317*	*3330*	O-H/N-H stretching
